# Dynamics of sperm transport and histological structure of the female reproductive system in *Octopus vulgaris* (Cephalopoda: Octopodidae)

**DOI:** 10.1371/journal.pone.0352331

**Published:** 2026-07-06

**Authors:** Hyeon Jin Kim, Jung Jun Park, Chang-Moon Lee, Jung Sick Lee

**Affiliations:** 1 Department of Aqualife Medicine, Chonnam National University, Yeosu, Republic of Korea; 2 Regional Leading Research Center, Chonnam National University, Yeosu, Republic of Korea; 3 Aquaculture Research Division, National Institute of Fisheries Science, Busan, Republic of Korea; 4 School of Healthcare and Biomedical Engineering, Chonnam National University, Yeosu, Republic of Korea; Laboratoire de Biologie du Développement de Villefranche-sur-Mer, FRANCE

## Abstract

In this study, we aimed to provide a detailed microanatomical description of the female reproductive system of *Octopus vulgaris*, an internally fertilized cephalopod, and to identify its functional characteristics. The female reproductive system of *O. vulgaris* consists of the ovary, common oviduct, proximal oviduct, oviducal gland, and distal oviduct, and histologically, the structure is similar to that reported in other octopods. The presence of spermatozoa and spermatophore remnants in the distal oviduct suggests that spermatophoric reaction may occur in this region. The released sperm then migrate to the spermathecae of the oviducal gland, where they are anchored to the epithelial layer via a helical acrosome and stored long-term with the support of secretory cells. During the spent and degenerative stages, sperm detach from the epithelial layer of the spermathecae in the oviducal gland and are released into the central cavity of the oviducal gland, where fertilization is presumed to occur. The fertilized eggs are then coated with secretory substances from the central and peripheral glands and subsequently pass through the acidic distal oviduct before being released. These findings demonstrate that the female reproductive system of *O. vulgaris* exhibits structural differentiation necessary for spermatophoric reaction, sperm storage, and fertilization. This study provides fundamental insights into the reproductive process of internally fertilized cephalopods and serves as a valuable reference for future research on cephalopod reproductive physiology.

## Introduction

In animals, fertilization is divided into external and internal fertilization depending on the fertilization site. Although external fertilization is common among aquatic animals, several groups including chondrichthyes [1], some teleosts [2–4], crustaceans [5], and cephalopods [6] exhibit internal fertilization.

Depending on the species, internal fertilization involves various reproductive strategies, including storage of the sperm received during copulation in the female reproductive system until fertilization. This has been reported in species that do not have simultaneous copulation and spawning, such as nematodes, annelida, insects, pisces, reptiles, amphibians, aves, mammals, cephalopods, and crustaceans [7–9]. Before fertilization, sperm storage extends the sperm lifespan, which enhances fertilization and reproduction rates [10].

In cephalopods, copulation occurs through the transfer of spermatophores from the male to the female via the hectocotylus, which is the third right arm of the male [11]. After being transferred to the female, the spermatophore is pulled by the cap thread through the action of the hectocotylus, inducing the spermatophoric reaction. Consequently, the spermatophore is released in the form of a spermatangium, in which the outer tunic has been removed [6]. The transferred spermatophore attaches to the female’s seminal receptacle or near the oviduct within the mantle cavity, serving as a storage site for sperm. The location and structure of sperm storage vary among species [6].

In octopod species, sperm storage conforms to the seminal receptacle type. However, unlike other cephalopods with the same type, the spermathecae are located within the oviducal gland [12,13]. In Octopodidae, spermatophores are known to be inserted into the female reproductive system via the hectocotylus [14]. However, reports on the spermatophoric reaction, sperm storage mechanisms, and fertilization site within the female reproductive system remain insufficiently detailed.

Among cephalopods, the female reproductive system of octopods, which is made up of an ovary, a pair of oviducal glands, and proximal and distal oviducts, is comparatively simple [15]. The microstructure of reproductive organs provides essential information for understanding species-specific reproductive strategies and for linking morphological characteristics with ecological adaptations. In Octopodidae, research on the female reproductive system has mainly focused on reproductive cycle and gonadal development [16–20], although recent studies have investigated the function and structure of the oviducal gland [21–24]. In contrast, structural changes in the oviduct associated with the reproductive cycle remain relatively poorly documented [25].

As an important species in Korean fisheries, understanding the reproductive biology of *Octopus vulgaris* is essential for effective resource management. In Korea, although *O. vulgaris* is one of the important species among cephalopods, octopus production continuously decreased from 10,813 tons in 2010, to 8,753 tons in 2015, and then to 8,283 tons in 2023 [26]. To conserve and manage fishery resources, it is important to have a precise understanding of their reproductive ecology. Therefore, this study aimed to investigate the microanatomical structure of the female reproductive organs in *O. vulgaris* and to examine structural changes across different gonadal developmental stages. Based on these observations, this study also aimed to provide insights into reproductive processes, including spermatophoric reaction, sperm storage, and fertilization within the female reproductive system. Furthermore, this foundational research seeks to provide insights into the reproductive ecological characteristics of internally fertilizing cephalopods.

## Materials and methods

### Sampling

**Fig 1:**
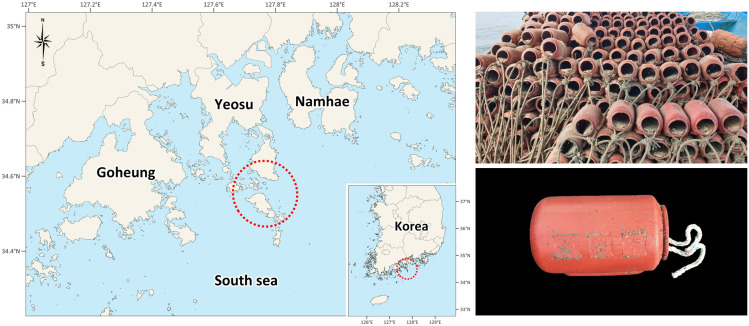
Sampling area and collection method (octopus pots) of *Octopus vulgaris.* Basemap data were obtained from V-World (https://www.vworld.kr/) and visualized using QGIS software. All photographs in this figure were taken by the authors.

*Octopus vulgaris* were collected using octopus pots in Yeosu (34°31’-34°35’N, 127°43’-127°47’E), on the southern coast of South Korea, from May 2022 to July 2023 (Fig 1). A total of 373 specimens (mean total weight 431.3 ± 238.8 g) were used for the analyses (about 25 specimens per month). All specimens were examined to determine ovarian developmental stage, and the female reproductive system of each individual was processed for histological analysis to evaluate structural and histochemical changes throughout the reproductive cycle.

All experimental procedures were conducted in accordance with the recommendations of the Institutional Animal Care and Use Committee of Chonnam National University (CNU IACUC-YS-2024-13). All individuals were anesthetized in 3% ethanol solution prepared in seawater until complete loss of responsiveness was observed. After deep anesthesia, animals were humanely euthanized by rapid dissection of the central nervous system prior to tissue sampling. The procedures followed established ethical guidelines and commonly used anesthesia protocols for cephalopod research [27].

### Light microscopy

For light microscopy, the female reproductive systems were dissected, fixed in aqueous Bouin’s solution, rinsed in running water, and subsequently dehydrated through a graded ethanol series (70%–100%). Sections were prepared according to the paraffin method using a microtome (Leica, Wetzlar, Germany). Sections were executed with Mayer’s hematoxylin-0.5% eosin (H-E) stain, Masson’s trichrome stain, alcian blue-periodic acid and Schiff’s solution (AB-PAS, pH 2.5) reaction, aldehyde fuchsin-alcian blue (AF-AB, pH 2.5) reaction.

### Scanning electron microscopy

Following the methodology reported in [28], a portion of the spermathecae in the oviducal gland was excised and prefixed in 2.5% glutaraldehyde buffered with 0.1 M phosphate (pH 7.4) for 3 hours. The fixed samples were washed three times for 20 minutes each with 0.1 M phosphate buffer (pH 7.4), dehydrated in an ascending series of alcohols (50%−100%), and then embedded in paraffin. The embedded specimens were sectioned into 3 µm thick sections using a microtome (Leica, Wetzlar, Germany). Then, the sections were placed on aluminum plates, paraffin was removed using xylene and the sections were dried. Afterwards, the surface was coated with platinum (10 mA/4 min) using a metal ion coater (SC7620, Quorum, UK) and observed using FE-SEM (Sigma 500, Zeiss, Germany) at an acceleration voltage of 1 kV.

### Transmission electron microscopy

Samples for transmission electron microscopy were prefixed in 2.5% glutaraldehyde buffered with 0.1 M phosphate (pH 7.4), followed by post-fixation at 4℃ using 1% osmium tetroxide. Next, they were washed with 0.1 M phosphate buffer (pH 7.4), followed by dehydration using ethanol. The samples were then polymerized into an epoxy resin using the substitution process with propylene oxide and then sectioned at a 60–90 nm thickness using an ultramicrotome (EM-UC 7, Leica, Germany), subjected to double-staining with uranyl acetate-lead citrate, and observed using FE-SEM (Sigma 500, Zeiss, Germany).

### Gonadal developmental stage

The gonadal developmental stage was classified into the following five stages based on the methods of [29]: 1) inactive stage, 2) early active stage, 3) late active and mature stage, 4) ripe stage, 5) spent and degenerative stage.

### Microscopic image analysis

For the quantification of each parameter at different stages of gonadal development, analyses were conducted using an image analyzer (i-Solution, IMT i-Solution Inc., Canada). The thickness of the epithelial layer was measured from the basal part of the epithelial cells to the free surface. For each gonadal developmental stage, 5–10 individuals were analyzed, and 40–50 measurements were taken per individual. The distribution of secretory cells was expressed as a percentage by measuring the areas of secretory cells and the epithelial layer and calculating them using equation (1).


$$\begin{array}{c} \text{Distribution of secretory cell }(\%)=\;\frac{\text{Secretory cell area }({\mu \text{m}}^{\text{2}})}{\text{Epithelial layer area }({\mu \text{m}}^{\text{2}})}\times \text{1}\text{0}\text{0} \end{array}$$ (1)

### Statistical analysis

Statistical analyses were performed using IBM SPSS Statistics software (IBM Corp., Armonk, NY, U.S.A.). Percentage data were arcsine square-root transformed prior to statistical analysis. Homogeneity of variances was evaluated using Levene’s test. When the assumption of equal variances was satisfied, one-way analysis of variance (ANOVA) followed by Tukey’s honestly significant difference (HSD) test was used for multiple comparisons. When the assumption of homogeneity of variances was violated, Welch’s ANOVA followed by the Games–Howell post hoc test was applied. Statistical significance was set at *p* < 0.05.

## Results

### Anatomy

**Fig 2:**
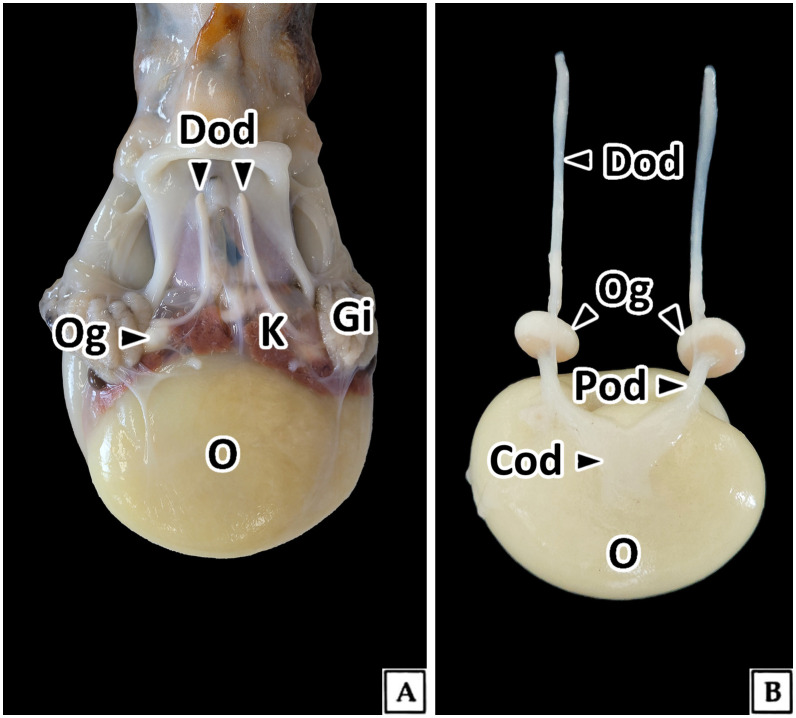
The anatomy (A) and morphology (B) of female reproductive system in *Octopus vulgaris.* Cod: common oviduct, Dod: distal oviduct, Gi: gill, K: kidney, O: ovary, Og: oviducal gland, Pod: proximal oviduct.

The internal organ systems of *Octopus vulgaris* occupy most of the mantle cavity. The ovary was located at the bottom of the mantle cavity, with a pair of brown kidneys right behind it and on its left. In the posterior part of the kidneys, there was a pair of gills on the left and right, as well as a light brown oval hepatopancreas (Fig 2A).

The female reproductive system, located in the lower part of the mantle cavity, consisted of the ovary, oviduct, and oviducal gland. The mature ovary was yellowish and round, and contained elongated oocytes. The oviduct, a milky–white tube, was divided into three parts: 1) the common oviduct, directly connected to the ovary, 2) the proximal oviduct, connected to the common oviduct, and 3) the distal oviduct, connected to the oviducal gland. The proximal oviduct was a branch of the common oviduct and connected to the paired oviducal gland, whereas the distal oviduct opened into the mantle cavity. The paired oviducal glands were oval-shaped, and their upper and lower parts were brown and milky white, respectively (Fig 2B).

### Histological structure

#### Distal oviduct.

**Fig 3:**
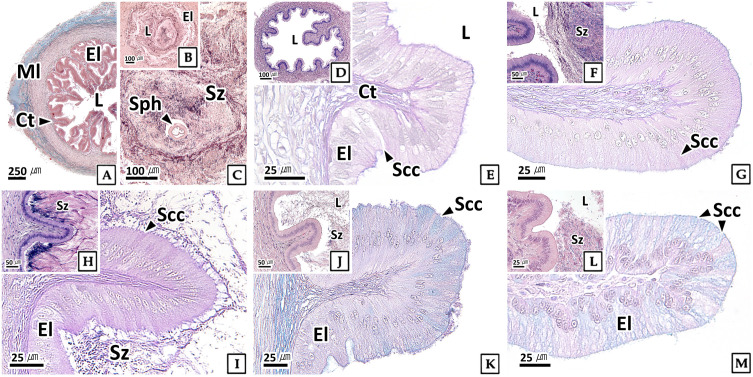
Distal oviduct of *Octopus vulgaris.* A: overall structure. B and C: showing the spermatophore remnants (Sph) and aggregates of spermatozoa (Sz) in the lumen **(L)**. D and E: inactive stage. F and G: early active stage. H and I: late active and mature stage. J and K: ripe stage. L and M: spent and degenerative stage. Ct: connective tissue, El: epithelial layer, Ml: muscular layer, Scc: secretory cell. A: Masson’s trichrome stain; B, C, D, F, H, J, L: H-E stain; E, G, I, K, M: AB-PAS (pH 2.5) reaction.

The distal oviduct consisted of a pair of tubular structures composed of three layers: an outer muscular layer, a connective tissue layer, and an epithelial layer, with multiple folds developed internally (Fig 3A). The muscular layer was a fibrous muscular layer composed of smooth muscle fibers and collagen fibers, while the connective tissue layer primarily consisted of loose connective tissue that formed the medullary part of the folds (Fig 3A). The epithelial layer was consisted of simple columnar epithelial cells and secretory cells. Although the secretory cells appeared as vacuoles in H-E stain, they showed a positive reaction in AB-PAS (pH 2.5) reaction, appearing blue. Spermatophore remnants and aggregates of spermatozoa were occasionally observed within the lumen of the distal oviduct (Fig 3B and C). From the early active stage to the spent and degenerative stage, a mixture of secretory substances and spermatozoa was observed within the lumen (Fig 3F-M).

**Fig 4:**
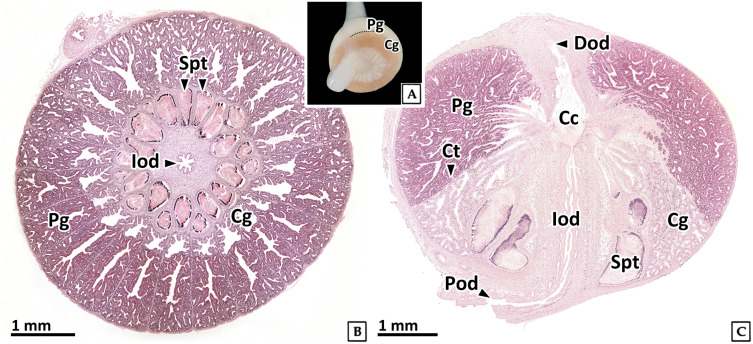
Distribution of secretory cell in the female reproductive systems with ovarian development stage of *Octopus vulgaris.* Values are presented as mean ± SD. Different letters indicate significant differences among ovarian developmental stages (*p* < 0.05). A: distal oviduct, B: central gland in oviducal gland, C: peripheral gland in oviducal gland, D: proximal oviduct. Ea: early active stage, In: inactive stage, Lm: late active and mature stage, R: ripe stage, Sd: spent and degenerative stage.

The distribution ratio of secretory cells showed an increasing trend throughout ovarian development and reached its highest mean value during the spent and degenerative stage. Significant differences were observed only between the inactive stage and the spent and degenerative stage (*p* < 0.05). However, acidic mucous-secreting cells were most abundant during the ripe stage, which corresponds to the period when fertilization primarily occurs (Figs 3 and 4A).

#### Oviducal glands.

**Fig 5:**
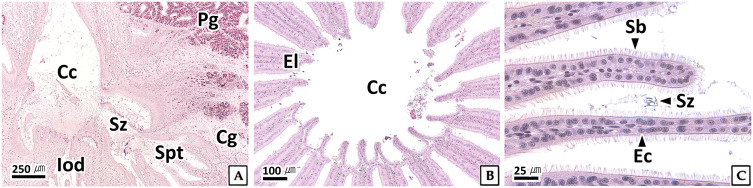
Central cavity (Cc) in the oviducal gland of *Octopus vulgaris.* A: longitudinal section. B and C: cross section. Cg: central gland, Ec: epithelial cell, El: epithelial layer, Iod: intraglandular oviduct, Pg: peripheral gland, Sb: striated border, Spt: spermathecae, Sz: spermatozoa. H-E stain.

In cross section, the oviducal gland was divided into the following concentric layers centered around the oviduct: 1) the spermathecae (seminal vesicles) in the inner layer, 2) the central gland in the middle layer, and 3) the peripheral gland in the outer layer, all of which merged into the central cavity located at the center of the oviducal gland (Fig 5).

**Fig 6:**
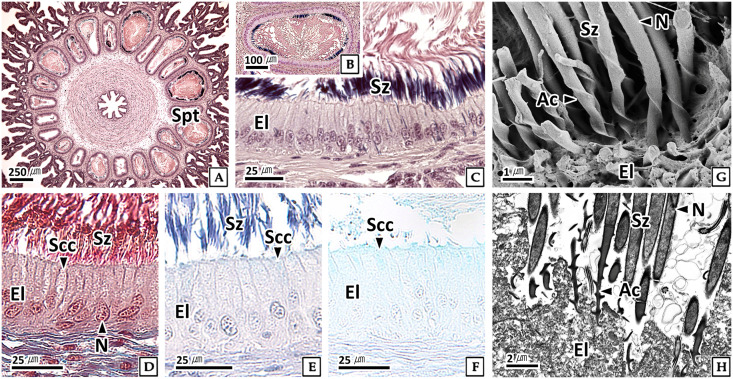
Spermathecae (Spt) in the oviducal gland of *Octopus vulgaris.* note that the acrosome (Ac) of the spermatozoa (Sz) is anchored in the epithelial layer (El). A-F: light micrographs. G: scanning electron micrograph. H: transmission electron micrograph. N: nucleus, Scc: secretory cell. A-C: H-E stain; D: Masson’s trichrome stain; E: AB-PAS (pH 2.5) reaction; F: AF-AB (pH 2.5) reaction.

The central cavity, which was located at the center of the oviducal gland, was connected to the spermathecae and distal oviduct, and it had a wrinkled structure composed of simple columnar epithelial cells with striated borders (Fig 6). During ovarian developmental stages, sperm were not observed in the inactive stage. However, from the ovarian early active stage to the spent and degenerative stage, numerous sperm and secretory substances were observed in the lumen.

**Fig 7:**
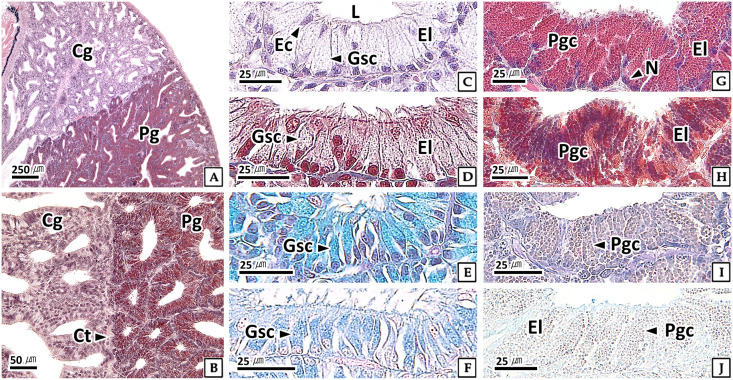
Proximal oviduct of *Octopus vulgaris.* A and B: overall structure, C: inactive stage, D: early active stage, E: late active and mature stage, F: ripe stage, G: spent and degenerative stage. Ct: connective tissue, Ec: epithelial cell, El: epithelial layer, L: lumen, Ml: muscular layer, Oc: oocyte, Sb: striated border, Scc: secretory cell. A: H-E stain, B: Masson’s trichrome stain, C-G: AB-PAS (pH 2.5) reaction.

**Fig 8:**
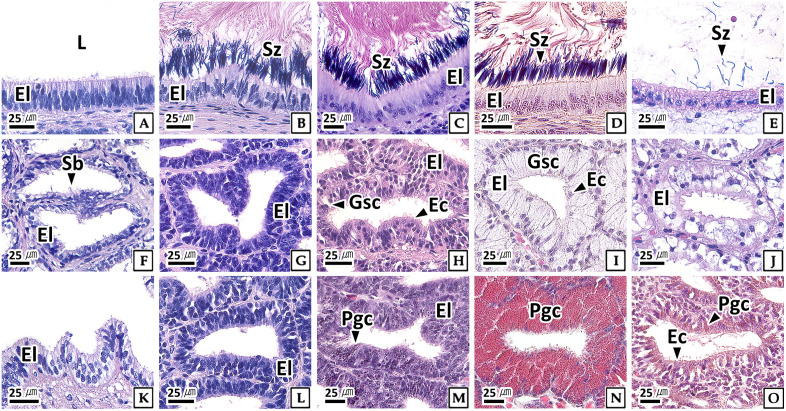
Oviducal gland with ovarian developmental stage of *Octopus vulgaris.* A-E: spermathecae. F-J: central gland. K-O: peripheral gland. A, F, K: inactive stage. B, G, L: early active stage. C, H, M: late active and mature stage. D, I, N: ripe stage. E, J, O: spent and degenerative stage. Ec: epithelial cell, El: epithelial layer, Gsc: glycoprotein-secreting cell, L: lumen, Pgc: proteinaceous granule-secreting cell, Sb: striated border, Sz: spermatozoa. H-E stain.

The spermathecae had about 20 chambers, each lined with a single layer of ciliated columnar epithelium and secretory cells (Fig 7A and 7B). In the secretory cells, cytoplasmic granules showed weak eosinophilic staining with H-E stain, red in Masson’s trichrome stain, and blue in AB-PAS (pH 2.5) and AF-AB (pH 2.5) reactions (Fig 7C–F). In the lumen, numerous sperm were observed adjacent to the free surface of the epithelial layer (Fig 7C–F). In H-E stain, the sperm head exhibited basophilic properties, while the tail showed eosinophilic (Fig 7C). Scanning and transmission electron microscopy revealed that the sperm head was anchored to the epithelial layer by a spiral-shaped acrosome, with the tail extending towards the lumen (Fig 7G and H). The structural changes in spermathecae at different ovarian developmental stages varied depending on whether sperm were anchored in the epithelial layer (Fig 8A-E). In the inactive stage, no sperm were observed (Fig 8A). However, from the early active stage to the ripe stage, sperm were densely packed and embedded in the epithelial layer (Fig 8B–D). In the spent and degenerative stages, only a few sperm remained anchored to the epithelial layer, while most were detached and dispersed into the lumen (Fig 8E).

**Fig 9:**
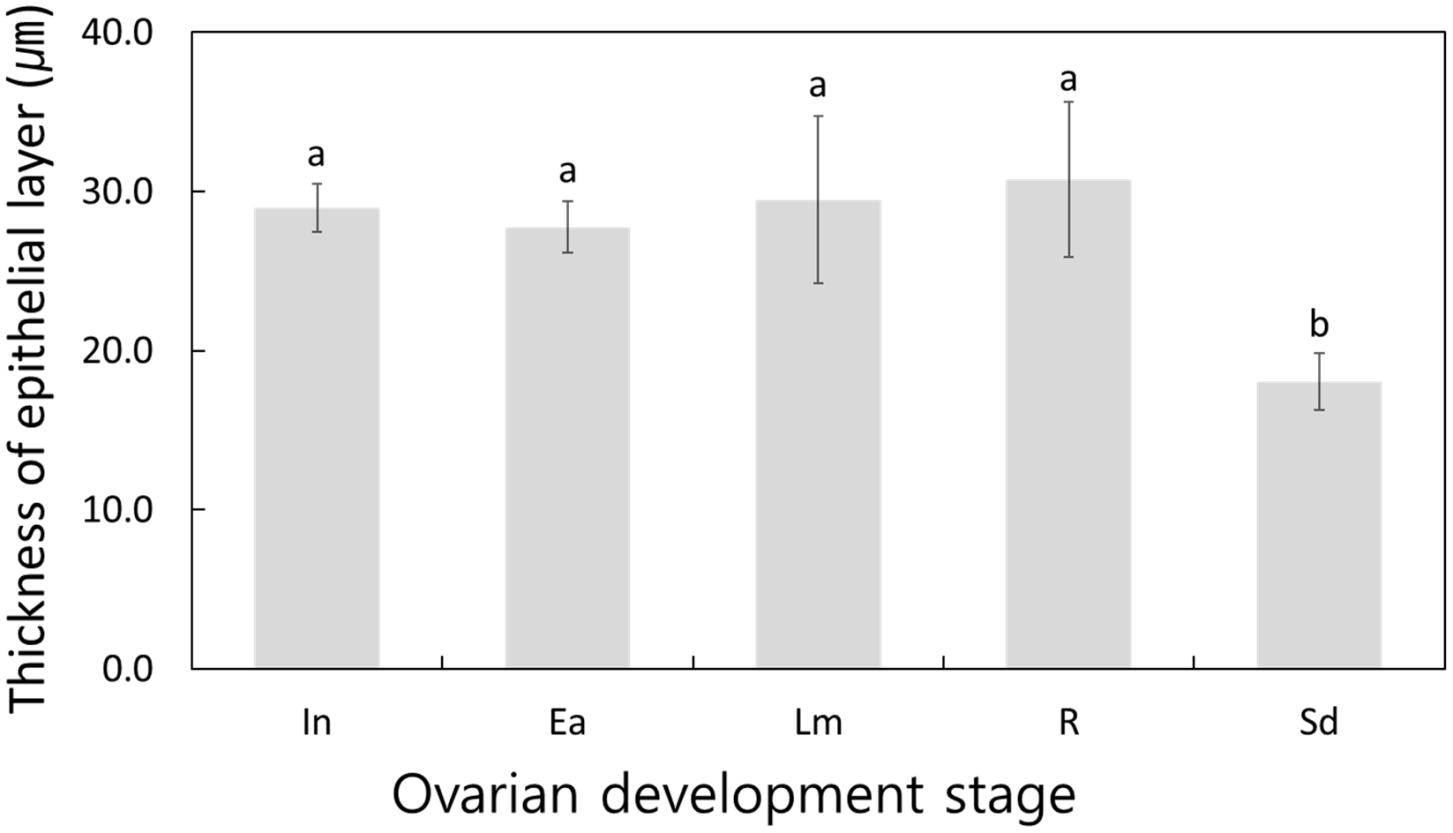
Thickness of epithelial layer of the spermathecae in the oviducal gland with ovarian development stage of *Octopus vulgaris.* Values are presented as mean ± SD. Ea: early active stage, In: inactive stage, Lm: late active and mature stage, R: ripe stage, Sd: spent and degenerative stage.

The epithelial layer thickness of spermathecae showed similar values, ranging from 27.8 to 30.7 μm, from the inactive stage to the ripe stage. In contrast, the thickness significantly decreased to 18.1 ± 1.8 μm in the spent and degenerative stages, showing a statistically significant difference compared to all other developmental stages (*p* < 0.05) (Fig 9).

**Fig 10:**
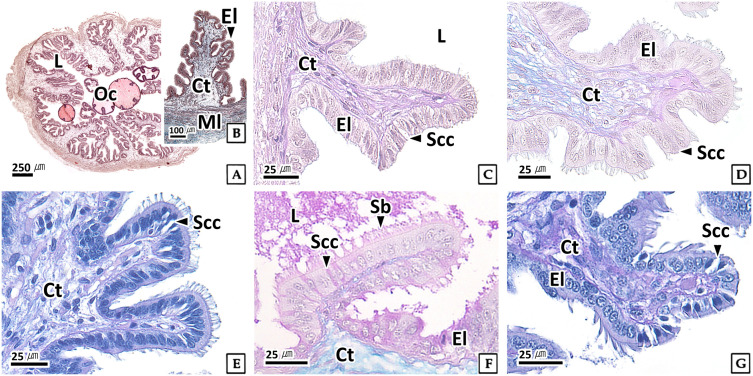
Central gland (Cg) and peripheral gland (Pg) in the oviducal gland of *Octopus vulgaris.* C-F: central gland, G-J: peripheral gland. Ct: connective tissue, Ec: epithelial cell, El: epithelial layer, Gsc: glycoprotein-secreting cell, L: lumen, N: nucleus, Pgc: proteinaceous granule-secreting cell. A-C, G: H-E stain, D and H: Masson’s trichrome stain, E and I: AB-PAS (pH 2.5) reaction, F and J: AF-AB (pH 2.5) reaction.

The central gland and the peripheral gland were separated by septa composed of connective tissue and consisted of a single epithelial layer composed of epithelial cells and secretory cells (Fig 10A and B). The two regions exhibited differences in the staining properties of their secretory cells. In the central gland, cytoplasmic granules in secretory cells were weakly basophilic in H-E stain and appeared red in Masson’s trichrome stain. They showed positive reactions to alcian blue in both AB-PAS (pH 2.5) and AF-AB (pH 2.5) reactions, appearing blue, indicating the presence of acidic nonsulfated carboxylated mucopolysaccharides (Fig 10C-F). In contrast, secretory granules in the peripheral gland were strongly eosinophilic in H-E stain and appeared red or purple in Masson’s trichrome stain. They appeared pale violet in AB-PAS (pH 2.5) reaction and light blue in AF-AB (pH 2.5) reaction, suggesting the presence of neutral carboxylated mucopolysaccharides (Fig 10G-J). Histologically, secretory cells in both glands became progressively more abundant with ovarian development and were markedly reduced during the spent and degenerative stage (Fig 8F-O).

The distribution of secretory cells in both the central gland and the peripheral gland was low during the inactive and early active stages, and increased significantly during the late active and mature stages. The highest values were observed during the ripe stage. Thereafter, the distribution decreased during the spent and degenerative stage, although this decrease was significant only in the peripheral gland (Fig 4B, C).

#### Proximal oviduct.

**Fig 11:**
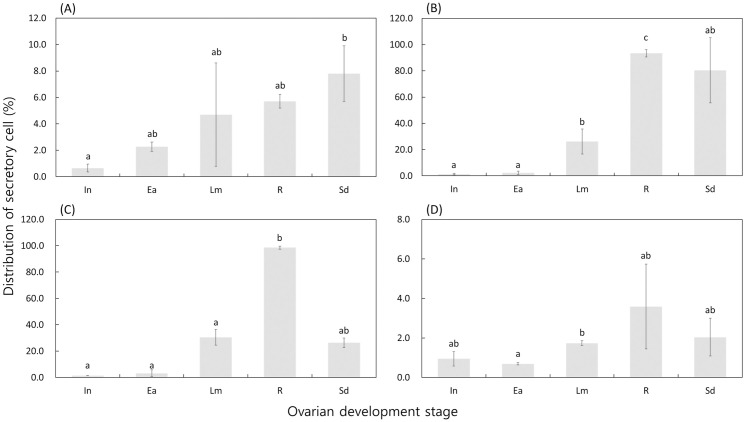
Oviducal gland of *Octopus vulgaris.* A: anatomical structure. B and C: light micrographs. B: cross section. C: longitudinal section. Cc: central cavity, Cg: central gland, Ct: connective tissue, Dod: distal oviduct, Iod: intraglandular oviduct, Pg: peripheral gland, Pod: proximal oviduct, Spt: spermathecae. H-E stain.

Like the distal oviduct, the proximal oviduct was divided into the muscular layer, connective tissue layer, and epithelial layer (Fig 11A). The muscular layer was composed of smooth muscle fibers and collagen fibers, while the connective tissue layer primarily consisted of loose connective tissue that formed the medullary part of the folds (Fig 11B). The epithelial layer consisted of a single layer of columnar epithelial cells and secretory cells. Secretory cells appeared as vacuoles in H-E stain and reddish–purple in AB-PAS (pH 2.5) reaction (Fig 11A and F). There were no structural differences in the proximal oviduct by ovarian developmental stage. In the ovarian ripe stage, mature oocytes were observed in the lumen, although sperm were not observed during any developmental stage (Fig 11C-G).

The distribution of secretory cells increased from the inactive stage (0.95%) to the ripe stage (3.59%) and subsequently decreased during the spent and degenerative stage (2.04%). Significant differences were observed only between the early active stage and the late active and mature stage (*p* < 0.05) (Fig 4D).

#### Ovary.

**Fig 12:**
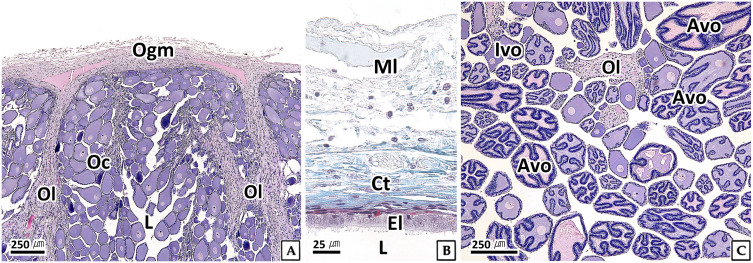
Ovary of *Octopus vulgaris.* Avo: active vitellogenic oocyte, Ct: connective tissue, El: epithelial layer, Ivo: initial vitellogenic oocyte, L: lumen, Ml: muscular layer, Oc: oocyte, Ogm: outer gonadal membrane, Ol: ovarian lamella. A and C: H-E stain; B: Masson’s trichrome stain.

The ovary was enclosed by a thin membrane composed of a muscular layer, connective tissue, and an inner epithelial layer. Inside, the ovarian lamella branched and wrinkled into several layers, with developing oocytes observed in the lumen (Fig 12A and B). Throughout the year, from the ovarian inactive stage to the spent and degenerative stage, sperm were not observed within the ovary (Fig 12C).

## Discussion

### Structure

In cephalopods, the female reproductive system varies with the order [30]. In decapods, the system is relatively complex and consists of an ovary, an oviduct, a paired nidamental gland, an accessory nidamental gland, and a seminal receptacle [30,31]. However, the octopods, *Octopus rubescens* [18], *O. mimus* [22], and *O. hubbsorum* [32], have an ovary, paired proximal oviducts, paired oviducal glands, and paired distal oviducts, distinguishing them from decapods.

The ovary can be classified into two types based on its outer membrane: the cystovarian type with a muscular outer membrane and the gymnovarian type with a connective tissue outer membrane. The ovary of *O. vulgaris* belongs to the cystovarian type and is composed of a muscular layer, connective tissue, and an epithelial layer. This cystovarian type has been reported in most teleosts [33–36] and cephalopods such as *O. hubbsorum* [32]. Although studies on the ovarian outer membrane in cephalopods remain scarce, the internal structure is similarly organized into multiple connective tissue–derived ovarian lamellae, as observed in the present study [25,37,38].

Generally, in octopuses, the proximal oviduct is directly connected to the ovary [15,39]. However, descriptions of a common oviduct are rare. In *O. hubbsorum*, a common oviduct has been reported outside the ovarian membrane [32]. In the present study, a similar Y-shaped structure was observed at the top, located externally to the ovarian membrane in *O. vulgaris*.

Among the reproductive systems, tubular organs like the oviduct play a role in transporting gametes (oocyte and sperm) and in moving reproductive products to the external environment. This transport is mediated by multiple mechanisms, including ciliary activity, muscular contractions, and secretory cells [40,41]. In this study, the proximal oviduct consisted of a simple epithelial layer composed of ciliated columnar epithelial cells and secretory cells, connective tissue layer, and muscular layer, a structure that is similar to that observed in *O. hubbsorum* [32] and *O.* vulgaris [25]. Additionally, the distribution of secretory cells was highest during the ripe stage in this study. This suggests that oocyte transport is facilitated not only by the increased number and surface area of epithelial folds and the presence of secreted substances in the lumen [25,42], but also through the lubricating function of the secreted substances, the ciliary movement of the epithelial layer, and the peristaltic motion of the fibrous muscular layer.

In general, in Octopodidae, the oviducal gland is composed of the spermathecae, the central gland, and peripheral gland. However, in *O. bimaculatus* [43], *O.* vulgaris [21], *Eledone cirrhosa* [44] and *O. mimus* [22], anatomical structures like the presence or absence of spermathecae, location and distribution of the gland, and the presence or absence of the central cavity, differ. This study found that in *O. vulgaris*, the oviducal gland was concentric, with the spermathecae on the inside, the central gland in the middle, and the peripheral gland on the outside. Additionally, in the center of the oviducal gland, a central cavity was connected to the spermathecae. A similar structure was reported for *Amphioctopus fangsiao* [24], *O. rubescens* [18], *O. vulgaris* [21,45], and *O. hubbsorum* [32].

The distal oviduct of *O. vulgaris* exhibited a structure similar to that of the proximal oviduct, and a comparable morphology was also reported in *O. rubescens* [18] and *O. hubbsorum* [32].

### Function: from sperm transport to spawning

In cephalopods, copulation occurs through the transfer of spermatophores to the female via the hectocotylus [11]. For sperm to be stored within the female reproductive system, the spermatophoric reaction, during which the spermatophore tunic is removed, must first occur. The spermatophoric reaction is triggered by factors such as osmotic pressure and contact with seawater [46,47].

In this study, spermatophore remnants and aggregates of spermatozoa were observed in the lumen of the distal oviduct, which is consistent with observations reported in *O. maya* [48]. Additionally, the presence of spermatophore remnants and numerous spermatozoa in the distal oviduct from the early active stage to the spent and degenerative stages suggests that the spermatophoric reaction occurs within the distal oviduct. However, the mechanism underlying this process remains unclear and requires further investigation. The released sperm are presumed to be transported toward the oviducal gland through ciliary movement of the epithelial layer and peristaltic contractions of the muscle fibers.

Internally fertilizing animals store sperm received from the male in the female’s sperm storage site [46]. The sperm storage site varies among species, and in most octopods, sperm are stored in the spermathecae within the oviducal gland [13]. Because sperm are stored in the female octopus until the female matures fully and ovulates [49], the sperm-storage site requires a mechanism or structure that supports successful sperm storage [50].

In octopods, sperm are stored in the spermathecae of the oviducal gland. In this study, the structure of the spermathecae was similar to previous reports [18,19,21,22,32,45]; however, a distinct feature was the presence of secretory cells within the epithelial layer. In octopods, sperm storage in the spermathecae is known to occur through anchoring of the helical acrosome to the epithelial layer [21,28,32], and in the present study, sperm of *O. vulgaris* were likewise observed to be anchored to the epithelial layer. Additionally, in *A. fangsiao*, sperm within the oviducal gland have been reported to be closely associated with ciliated columnar epithelial cells or entangled in secretory substances within the lumen of the spermathecae [24]. In the present study, sperm were observed to remain attached to the epithelial layer of the spermathecae from the early active stage to the ripe stage, indicating that sperm can be stored for an extended period within the female reproductive system. During the subsequent spent and degenerative stages, sperm were released from the spermathecae into the lumen, accompanied by a reduction in epithelial thickness. These findings suggest that secretory cells of the epithelial layer may contribute to supplying nutrients and energy that support long-term sperm storage [24,51]. As sperm exit the spermathecae for fertilization, the epithelial layer loses its function, leading to a reduction in thickness due to atrophy.

In cephalopods, fertilization is divided into external and internal fertilization. External fertilization occurs in most cephalopods, such as squid and cuttlefish [6], whereas internal fertilization is mainly observed in octopods. Even among octopods, however, the fertilization site varies among taxa.

Octopods such as *Eledone massyae* and *E. gaucha*, as well as pelagic species in the families Argonautidae, Alloposidae, Ocythoidae, and Tremoctopodidae, lack spermathecae in the oviducal gland, and sperm are instead stored in the ovarian cavity for fertilization [44,52]. In *O. mimus*, spermathecae are present within the oviducal gland, but the absence of a central cavity results in fertilization occurring in the interglandular oviduct [22]. By contrast, most octopuses, with the exception of *O. bimaculatus* [43] and *Eledone cirrhosa* [44], have spermathecae in the oviducal gland, where sperm are stored for fertilization [13,21,39,53]. In *O. vulgaris* [21] and *A. fangsiao* [24], when the oocyte reaches the central cavity, fertilization occurs in the central cavity of the oviducal gland via sperm release from the spermathecae. In the present study, sperm were attached to the free surface of the epithelial layer within the spermathecae from the early active stage to the ripe stage. In the subsequent spent and degenerative stage, sperm detached from the epithelial layer and were observed within the lumen of the central cavity. These findings suggest that in the female reproductive system of *O. vulgaris*, sperm are stored in the spermathecae of the oviducal gland after copulation and subsequently released into the central cavity during ovulation, where fertilization is presumed to occur.

Once fertilization occurs in the central cavity, the fertilized eggs are stabilized and attached by secretory substances produced by the oviducal gland [21,39,54]. Although previous studies have reported variations in staining properties, it is generally recognized that the central gland secretes mucopolysaccharides, whereas the peripheral gland secretes mucoproteins [21,39,53,55]. In the present study, the central gland was found to contain acidic carboxylated mucopolysaccharides, while the peripheral gland contained neutral carboxylated mucopolysaccharides, consistent with previous studies. Furthermore, the distribution of secretory cells in both glands peaked during the ripe stage and declined in the spent and degenerative stages.

Fertilized eggs in the oviducal gland are released through the distal oviduct and are coated by substances secreted from the central and peripheral glands. Previous studies reported that the pH of the distal oviduct in *O. vulgaris* is approximately 5.2 in mature individuals and 6.8 in immature individuals, and that egg coating remains stable within a pH range of 5.5–9.0 but dissolves outside this range [21]. This pH reduction has been attributed to the activity of mucous cells in the distal oviduct [43]. In the present study, acidic mucous secretory cells were most abundant during the ripe stage. These results are consistent with previous studies suggesting that activation of acidic mucous secretory cells may contribute to reduction of pH in the distal oviduct. Such regulation of the luminal pH may inhibit egg coat polymerization prior to spawning, thereby allowing fertilized eggs to pass normally through the reproductive tract.

This study described the histological structure of the female reproductive system of *O. vulgaris* and the changes associated with ovarian development. The structural and histochemical characteristics of the female reproductive system, together with the distribution of sperm within the reproductive system, support its involvement in spermatophoric reaction, sperm storage, and fertilization. These findings provide further insight into the reproductive biology of this species.
